# Acquired tumor cell resistance to sunitinib by increased invasion and epithelial-mesenchymal transition in LL/2 murine lung cancer

**DOI:** 10.18632/oncotarget.19295

**Published:** 2017-07-17

**Authors:** Yang Du, Jia-Qi Liu, Jie Tang, Jun Ge, Ye Chen, Ke Cheng, Jing Ding, Zhi-Ke Li, Ji-Yan Liu

**Affiliations:** ^1^ Department of Medical Oncology, Cancer Center, State Key Laboratory of Biotherapy, West China Hospital, West China Medical School, Sichuan University, GuoXue Xiang, Chengdu 610041, Sichuan Province, China

**Keywords:** sunitinib, drug-resistance, lung cancer, increased invasion, epithelial-mesenchymal transition

## Abstract

**Objective:**

This study aims to investigate biological behavior changes in a murine lung cancer cell characterized by acquired resistance to sunitinib, a potent inhibitor of multiple-targeted receptor tyrosine kinase.

**Methods:**

A lung cancer cell line resistant to sunitinib (LL/2-R) was developed from its parental cell line (LL/2-P). Differences in biological characteristics and associated molecular profiles between these two cells were compared *in vitro* and *in vivo*.

**Results:**

LL/2-R cells showed an approximately 5-fold higher IC_50_ of sunitinib than LL/2-P cells and exhibited a reduced growth inhibition following sunitinib treatment compared with LL/2-P. In LL/2-R cells and tumors, increased migration, invasion and metastasis were observed, along with upregulation of MMP-2 and MMP-9. We also analyzed the molecular profiles involved in EMT, and found that E-cadherin was downregulated in LL/2-R tumors, and vimentin was upregulated in LL/2-R cells and tumors, along with β-catenin translocating to the nuclei in LL/2-R cells. Furthermore, transcriptional factors mediated EMT, snail and twist, and the secretion of TGFβ1 also increased in LL/2-R cells and tumors.

**Conclusions:**

We established a sunitinib-resistant lung cancer cell line and confirmed its drug-resistance to sunitinib *in vivo*. Our results implied that increased invasion and EMT may associate with the acquisition of resistant phenotype to sunitinib in cancer cells.

## INTRODUCTION

Sunitinib, an oral small molecular multipletarg-eted receptor tyrosine kinase (RTK) inhibitor, has a relatively board spectrum of targets, including the vascular endothelial growth factor receptors (VEGFRs); platelet-derived growth factor receptors (PDGFRs); the stem cell factor receptor (c-KIT) and FMS-like tyrosine kinase 3(FLT3) [1–4], thereby exhibiting both anti-angiogenesis and anti-tumor activities. In the clinic, sunitinib has benefited many patients with advanced renal cell carcinoma (RCC), imatinib-refractory gastrointestinal stromal tumor (GIST) or pancreatic neuroendocrine tumor (pNET) [5–7]. Additionally, clinical data and experimental evidence suggested that sunitinib also has demonstrable efficacy on other solid tumors, such as lung cancer [8, 4, 9, 10].

However, future of sunitinib is facing a major challenge: an emergent resistance to sunitinib will eventually develop. Generally, two groups of underlying mechanisms of resistance have been elucidated: first, tumor cells themselves mediate resistance to sunitinib. For instance, a study uncovered that continued exposure to sunitinib could cause an increased lysosomal capacity in tumor cells, resulting in resistance to sunitinib [11]. Second, the other mechanisms mainly attribute to tumor microenvironment. Finke et al elaborated that myeloid derived suppressor cells (MDSCs) induced sunitinib-resistance via providing enduring angiogenesis and immune suppression [12]. In this study, we focused on the biological behavior changes happened to sunitinib-resistant tumor cells and its possible explanation for sunitinib-resistance.

In the attempt of addressing these questions, we developed a sunitinib-resistant lung cancer cell line *in vitro*, and then confirmed its drug-resistance to sunitnib *in vivo*. Moreover, we further investigated the changes in biological behavior and molecular profiles both *in vitro* and *in vivo*.

## RESULTS

### Establishment of acquired resistant LL/2 cell line to sunitinib

**Figure 1:**
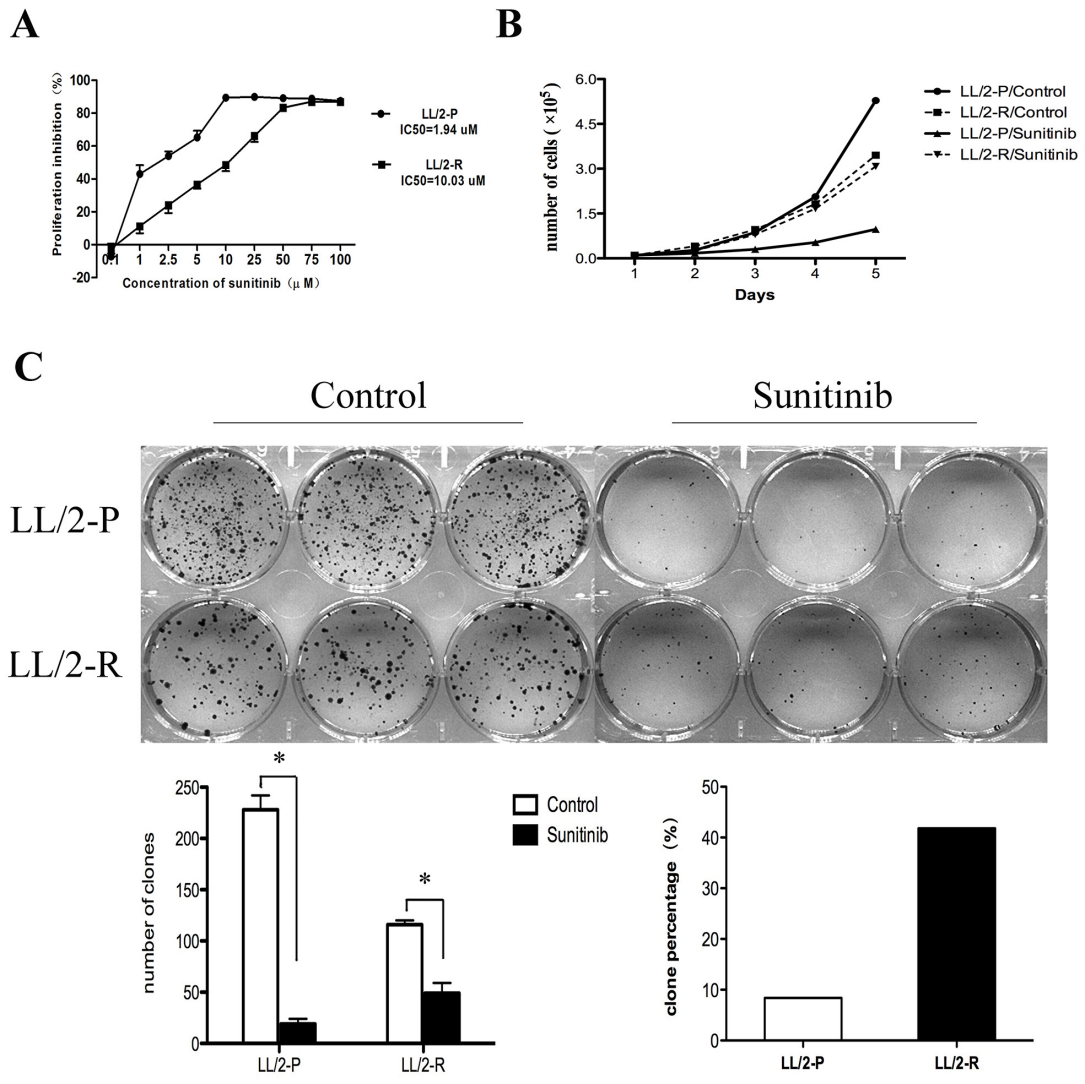
Acquired resistance to sunitinib in LL/2-R cells To induce resistance, LL/2 cell line was continuously exposed for more than 6 months to gradually increasing concentrations of sunitinib. **(A)** Value of IC_50_ to sunitinib increased in LL/2-R cells. Points, mean of three independent experiments; bars, SD. **(B)** LL/2-R cells had a reduced cell growth inhibition, when treated with sunitnib. **(C)** LL/2-R cells had an increased clonogenic capacity, when treated with sunitinib. Cells were exposed to 1μm sunitinib for 10 days, and each group is made in triplicate. Columns, mean of three independent experiments; bars, SD; *, P<0.05.

LL/2-P cells were treated with sunitinib for more than 6 months at gradually increasing concentrations. Finally, sunitinib-resistant LL/2 cell line, LL/2-R, was established. The values of IC_50_ for the LL/2 cell sublines were determined by MTT. The IC_50_ of LL/2-R cell line showed about 5-fold higher concentrations compared to that of LL/2-P cell line: 10.03μM vs.1.94μM (Figure 1A).

In addition, we found that LL/2-R cells had a reduced cell proliferation inhibition and an increased clonogenic capacity compared to LL/2-P cells, when treated with sunitinib. In cell proliferation assay, the doubling time (DT) of LL/2-P cell line was 36.2h and 19.7h in sunitinib- and vehicle- treated cells, respectively, while it was 26.7h and 25.5h for LL/2-R cell line correspondingly. The proliferation inhibition rate was 85.2% in LL/2-P cell line, but only 13.8% in LL/2-R cell line (Figure 1B). In clonogenic assay, the number of clonies in LL/2-R cell line was 49.0±10.0 and 116.0±4.0 in sunitinib- and vehicle-treated wells, respectively, while it was 19±5 and 228±14 in LL/2-P cell line. The percentage of clony formation in LL/2-R cell line was 41.8%, but only 8.3% in LL/2-P cell line (Figure 1C). In conclusion, LL/2-R cells had a higher resistance to sunitinib treatment in contrast to LL/2-P cells *in vitro*.

### Decreased growth-inhibitory effect of sunitinib on LL/2-R cells *in vivo*

**Figure 2:**
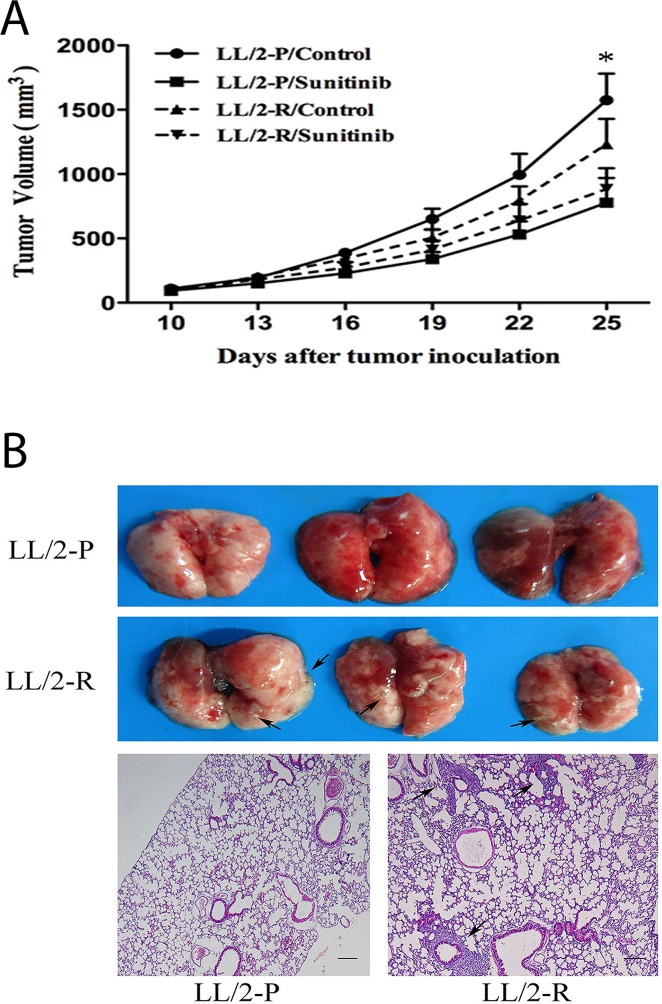
Decreased growth-inhibitory effect of sunitinib and enhanced metastatic potential of LL/2-R cell subline *in vivo* **(A)** Decreased growth-inhibitory effect of sunitinib on LL/2-R cells *in vivo.* Growth curve of tumors established from LL/2-P and LL/2-R tumors after tumor cells injection (5×10^5^ cells, n=10). Mice received treatment with vehicle or sunitinib (80mg/kg/day) 10 days after tumor cells injection. Points, mean of three independent experiments; bars, SD; *, P<0.05. **(B)** Increased metastatic potential of LL/2-R cells *in vivo*. 40 days after LL/2 cell sublines subcutaneous injection (5×10^5^ cells, n=3), lungs in mice were examined and analyzed by histological H&E staining of tissue sections. Metastases were observed, as indicated by the black arrows. Representative images are shown. Scale bars, 200μm(×40).

To verify whether LL/2-R cells also had sunitinib-resistance *in vivo*, LL/2-R cells and LL/2-P cells were injected subcutaneously in the right flank of mice, respectively. Ten days after injection, tumors were established (about 100mm^3^ in size) and treatment with sunitinib (80mg/kg/day) or vehicle was performed. Sunitinib resulted in a growth inhibition of LL/2-R tumors by 28.2% and LL/2-P tumors by 51.3% (Figure 2A). We confirmed that LL/2-R cells were also more resistant to sunitinib treatment *in vivo*, compared to LL/2-P cells.

### Increased migratory, invasive and metastatic potential of LL/2-R cell subline

**Figure 3:**
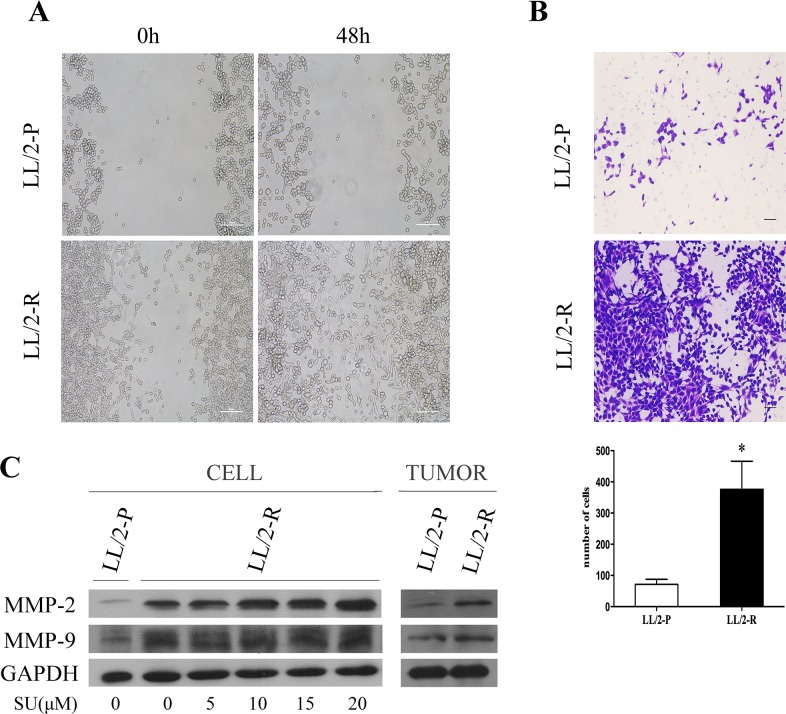
Increased migratory and invasive potential of LL/2-R cell subline **(A)** A wound healing assay showed that LL/2-R cells had increased migratory ability. Representative images are shown imediately after a scratch was created (0h) and 48h later. Scale bars, 100μm (×100). **(B)** LL/2-R cells had increased ability to invade through Matrigel-coated transwell membranes. After 48 hours, the invaded cells were stained, photographed and counted. Representative photographs of transwell membranes showed stained invaded cells. Columns, mean of three independent experiments; bars, SD; *, P<0.05; scale bars, 50μm(×100). **(C)** Western blotting assay was used to investigate MMP-2 and MMP-9 levels of LL/2 cells and tumors, with GAPDH as a loading control. The MMP2 and MMP9 levels of LL/2-R cells and tumors were upregulated, compared with LL/2-P. The expression of MMP2 and MMP9 in LL/2-R cells seems increased when treated with climbing concentration of sunitinib for 48h from 5μM to 20μM. Three independent experiments were conducted.

The cell migration was analyzed by wound healing assay. By 48h post-wounding, LL/2-R cells significantly improved closure of wound compared to LL/2-P cells, showing a faster migration (Figure 3A). The invasive capacity of LL/2 cell sublines was examined by transwell assay. The numbers of LL/2-R and LL/2-P cells that invaded through the basement membrane were 376.0 ± 90.0 and 71.5 ± 16.2, respectively (Figure 3B). To further investigate the metastatic potential of LL/2 cell sublines *in vivo*, LL/2-R cells and LL/2-P cells were injected subcutaneously in the right flank of mice, respectively. 40 days later, lung metastases could be detected on mice bearing LL/2-R tumors, but not on mice bearing LL/2-P tumors (Figure 2B). In addition, MMPs has been reported to play crucial roles in invasion and metastasis of tumor cells, thus protein levels of two of most important MMPs, MMP-2 and MMP-9, in LL/2-R and LL/2-P cells and tumors were measured by western blotting assay. The expression of MMP-2 and MMP-9 were both higher in LL/2-R than LL/2-P cells (Figure 3C), also higher in tumor tissues from LL/2-R than LL/2-P in mouse models. The data indicated that LL/2-R cells possessed increased migratory, invasive and metastatic potential.

### EMT characteristics of LL/2-R cell

**Figure 4:**
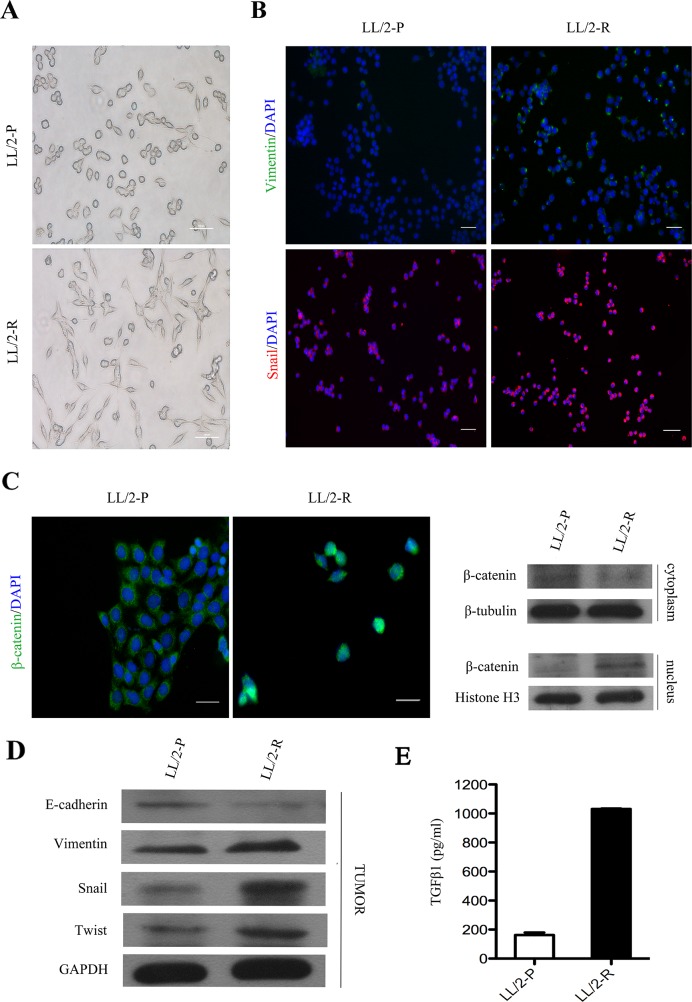
EMT of LL/2-R cell subline both *in vitro* and *in vivo* **(A)** The morphology of LL/2-P and LL/-R cells. Scale bars, 100μm(×100). **(B)** Immunofluorescence images of LL/2 cell sublines. First row, vimentin imunolocalisation (green colour) was detectable in LL/2-R cells, but absent from LL/2-P cells. Second row, snail imunolocalisation (red colour) in nucleus was stronger in LL/2-R cells. Cell nuclei are coloured blue after staining with DAPI. Scale bars, 100μm (×100). **(C)** Nuclear translocation of β-cateninin LL/2-R cells. Immunofluorescence images displayed that β-catenin (green colour) gave nuclear foci pattern of imunolocalisation in LL/2-R cells, but diffuse cytoplasmic pattern in LL/2-P cells. Western blotting also showed that the β-catenin level of nucleus was upregulation, but that of cytoplasm was downregulation in LL/2-R cells. Cell nuclei are coloured blue after staining with DAPI. Scale bars, 50μm(×400). β-tubulin and Histone H3 were used as loading controls respective for cytoplasmic and nuclear protein. **(D)** Alterations in EMT marker in LL/2-R tumors. Western blotting revealed that E-cadherin was downregulation, and vimentin, snail and twist were all upregulation, with GAPDH as a loading control in LL/2-R tumors. Three independent experiments were conducted. **(E)** The TGFβ1 level was significantly increased in LL/2-R cells, which is measured by ELISA assays.

It has been reported that epithelial-mesenchymal transition (EMT) is associated with increased invasiveness of tumor cells. Firstly, we found that LL/2-R cells changed to flat and spindle-sharped, showing a mesenchymal morphology (Figure 4A). Therefore, we investigate expression of vimentin and snail, and location of β-catenin in LL/2 cell sublines *in vitro*. The immunofluorescence staining showed that the expression level of vimentin and snail increased in LL/2-R cells relative to LL/2-P cells, while the immunofluorescence staining and western blotting assay both confirmed that β-catenin translocated to the nucleus (Figure 4B and 4C). Additionally, we further tested whether LL/2-R cells also undergone EMT *in vivo* using western blotting assay. The expression of E-cadherin, an epithelial cell marker, significantly decreased, conversely, the protein level of vimentin, a mesenchymal cell marker, increased in LL/2-R tumors. Next we further analyzed the expression of EMT-related transcriptional factors like snail and twist. They were both upregulated in LL/2-R tumors (Figure 4D). As one of the main player in inducing EMT, the TGFβ1 secretion of LL/2-R cells was also remarkably increased, compared to LL/2-P cells (Figure 4E). Our results demonstrated that LL/2-R cells undergone EMT.

## DISCUSSION

Tyrosine kinase inhibitors (TKIs), such as sunitinib, are the major targeted therapies for many cancers. Unfortunately, resistance to the TKIs will eventually develop. To study the mechanism, we developed a resistant lung cancer cell and animal model to sunitinib, which have been tested in several clinical trials and preclinical studies in lung cancer [13–15], and subsequently validated the resistance to sunitinib both *in vitro* and *in vivo*. Our results that acquired tumor cell resistance to sunitinib causes resistance *in vivo* support the concept that tumor cells themselves might play a crucial role in resistance to sunitinib.

Thus, we focused our investigation on the changes in biological characteristics of tumor cells, as several studies also pointed that a significant role for tumor cells themselves in development of drug-resistance [16–18]. We observed that sunitinib-resistant LL/2 cancer cells exhibited increased migration and invasion *in vitro*, and enhanced metastatic potential *in vivo*, companied by MMP2 and MMP9 overexpression, which play crucial roles in tumor invasion and metastasis [19, 20]. Similarly, there have been previous links reported between drug-resistance and increased invasion [21–23]. Du et al [24] and Paez-Ribes et al [25] also showed that more invasive behavior was observed in cancer cells which are resistant to antiangiogenic therapy. Our data and studies implicate that invading more aggressively into normal tissue is possibly another latent adaption mode of tumor cells.

To invade into normal tissue, tumor cells have to dissolve cell-cell junction and cell-ECM adhesion. As a process during which the polarized, stationary epithelial cells break down their cell-cell and cell-ECM contacts and convert intosingle dissociated, non-polarized, motile mesenchymal cells [26], the EMT has been reported that associated with cancer cell invasion and distant metastasis [27, 28]. When cancer cells undergo EMT, some molecular changes occur. For example, E-cadherin, a typical marker of epithelial cells, is lost, on the other hand, vimentin, a typical maker of mesenchymal cells, is induced [29]. Besides, E-cadherin is anchored to β-catenin at the epithelial cell membrane to maintain integrity of cell-cell adhesion junctions (AJs). Therefore, loss of E-cadherin also results in nuclear translocation of β-catenin, inducing activation transcriptional factor LEF/TCF4 which facilitates EMT by activating Wnt signaling pathway [30]. Moreover, EMT is driven by transcriptional factors that repress the expression of E-cadherin, such as snail and twist [31, 32]. In this study, we observed morphology changes, downregulation of E-cadherin, on the contrary, upregulation of vimentin, snail and twist, along with nuclear translocation of β-catenin, in LL/2-R cells. Based on these results, we believe that sunitinib-resistant LL/2 cancer cells may experience EMT, which results in their increased activity to invade. Besides, we detected that the TGFβ1 level of LL/2-R cells was dramatically increased, and previous studies had reported that TGFβ1 could induce EMT. So the EMT process occurred in LL/2-R cells may be TGFβ1-dependent.

In the present study, we described that LL/2-R cancer cells undergone EMT, along with their resistant phenotype. To date, there is growing acceptance that sunitinib-resistance and EMT have strictly connection, as we displayed in our work as well. The EMT phenomena have been observed in some other sunitinib-resistant cancers, such as prostate cancer, hepatocellular carcinoma, renal cell carcinoma [33, 34]. Hammers et al. also described that EMT may be correlated with acquired resistance to sunitinib in patients with clear cell renal carcinoma [35]. Yet, the underlying mechanism between sunitinib-resistance and EMT remains unclear. Previous studies deemed that the EMT is triggered by hypoxic tumor microenvironment, which is induced by anti-angiogenesis therapy, in sunitinib-resistant cancers. However, we found that EMT characteristics also could be observed in sunitinib-resistant cancer cells *in vitro* in normoxia, meaning that sunitinib has a direct impact on tumor cells, eventually, EMT occurs. A previous study had reported that sunitinib could remarkly induced the expression of TGFβ [36], which is in accordance with our finding. These results suggested that sunitinib could induce the upregulation of TGFβ, which might be associated with EMT. Further research is needed to figure out the underlying mechanisms.

In conclusion, we successfully established LL/2-R cell line, which exhibits decreased sensitivity to sunitinib as opposed to its parental cell line, both *in vitro* and *in vivo*. We subsequently showed that resistant cells are possessed of increased invasive capacity and enriched EMT properties, which may be involved in acquisition of a phenotype resistant to sunitinib in LL/2 cells and this EMT maybe TGFβ1-dependent. Thus, our results warrant further studies to investigate the mechanism of resistance and promising therapeutic strategies based on circumvention of EMT during sunitinib treatment.

## MATERIALS AND METHODS

### Cell culture and reagents

The lewis lung carcinoma cell LL/2 was obtained from ATCC (American Tissue Culture Collection) and cultured in Dulbecco’s Modified Eagle’s Medium (DMEM) supplemented with 10% FBS and maintained in a humidified incubator containing 5% CO2 at 37°C. Sunitinib malate was purchased from Selleckchem. For the development of sunitinib-resistant subline LL/2-R, the parental cell line LL/2-P was continuously exposed for more than 6 months to gradually increasing concentrations of sunitinib, which is increased by 0.2μm every 48h until the IC_50_ to 2μm, and then by 0.5μm until to 20μm.

### MTT assay

Cells (2×10^3^ per well) were seeded in 96-well plates, and then incubated with different concentrations of sunitinib on the next day. 48hours later, the metabolically active cells were quantified using MTT (5mg/ml, Sigma-Aldrich) by measuring the Optical Density (OD) value at 570nm in ELISA reader. The proliferation inhibition due to different concentration of sunitinib was calculated by the following formula: proliferation inhibition (%)=(OD_treated_-OD_control_)/(OD_control_-OD_blank_)×100. The value of IC_50_ (the concentration required for a 50% proliferation inhibition) was determined by Graphpad prism 5.0.

### Cell proliferation assay

Cells (5×10^3^ per well) were seeded in 24-well plates. After 24 hours, culture medium was replaced with fresh medium or that containing 2μM sunitinib. The cells were counted daily in triplicate by Trypan blue dye exclusion assay. The values of doubling time (DT) and proliferation inhibition were calculated, according to the following formulas: DT(h)=[lg2/(lgNt-lgN_0_)]×t (Nt = ultimate cell number; N_0_ = primary cell number; t = termination incubation time) and proliferation inhibition (%)=(N_0_-N)/N_0_×100 (N_0_=number of cells in untreated well; N=number of cells in treated well).

### Clonogenic assay

Cells (1×10^3^ per well) were exposed to 1μm sunitinib for 10 days to allow clony formation. Clonies were fixed, stained with crystal violet and counted manually, with a minimal clony cells number of 50 for required counting. The percentage of clony formation is calculated as the number of clonies in treated wells divided by those obtained in untreated wells.

### Wound healing assay

Cells (2×10^6^ per well) were seeded in 6-well plates. Scrape the cell monolayer in a straight line to create a scratch with a p200 pipet tip. To obtain the same field during the image acquisition, reference points was made on the outer bottom of the dish. Sequentially, the images were captured with a Nikon Eclipse Ti-U inverted microscope at 0h and 48h, respectively.

### Transwell invasion assay

Cells were starved in serum-free DMEM for 48h. Transwell chambers (Corning Costar) consisting of 8μm pore size membrane filter inserts were coated with matrigel (BD Bioscience) overnight in incubator. 1×10^5^ cells resuspended in serum-free medium were placed in the upper chamber, and medium supplemented with 10% FBS were added into the lower chamber. After 48 hours, cells on the upper surface of the membrane were removed with a cotton swab, and cells on the lower surface were stained with crystal violet, photographed and counted using an Olympus BX51 microscope.

### Immunofluorescence assay

Cells on coverslips were fixed with 4% paraformaldehyde, permeabilized in 1% (V/V) Triton X-100 in PBS, blocked with 5% BSA and incubated with primary antibodies at 1:100 dilution at 4°C overnight. Secondary FITC-conjugated anti-rabbit or Texas Red-conjugated anti-mouse antibody (Santa Cruz Biotechnology) was used at 1: 50 dilution, and then DAPI (Beyotime) was included in this incubation for last 10 min. Coverslips were photographed with a Nikon Eclipse Ti-U inverted fluorescence microscope. Primary antibodies used are as follows: mouse anti-snail (Merck Millipore), rabbit anti-vimentin and rabbit anti-β-catenin (Cell Signaling Technology).

### Western blotting assay

Total protein samples were (30μg) separated by SDS polyacrylamide gel and then transferred to PVDF membrane. After blocking with 5% (W/V) nonfat-dried milk, membranes were incubated with primary antibodies at 1:1000 dilution at 4°C overnight, followed by incubation with the appropriate HRP-conjugated secondary antibodies (ZSGB-BIO) at 1:10000 dilution. The immune complexes were detected by an enhanced chemiluminescence system and exposed on Kodak X-ray films. Primary antibodies used are as follows: mouse anti-GAPDH (Beyotime), rabbit anti-MMP-2, rabbit anti-MMP-9, rabbit anti-E-cadherin, rabbit anti-vimentin, rabbit anti-β-catenin, rabbit anti-Histone H3 (Cell Signaling Technology), mouse anti-β-tubulin (Sigma-Aldrich), rabbit anti-snail and rabbit anti-twist (Zen Bioscience).

### Measurement of TGFβ1 level

Cells (2×10^6^ per well) were seeded in 6-well plates. 48 hours later, the TGFβ1 level in cultured medium was quantified using the Mouse TGFβ1 ELISA kit (Neobioscience) in accordance with manufacturer’s protocol.

### Tumor growth *in vivo*

All animal experiments were performed according to procedures approved by the Institutional Animal Use and Care Committee of Sichuan University, China. C57BL/6 mice were purchased from HuaFukang Biological Technology Co. Ltd. (Beijing, China) and housed under SPF (specific pathogen-free) conditions. Tumor cells (5×10^5^ cells per mouse for LL/2-P or LL/2-R) were injected subcutaneously in the right flank of mice. When tumor size reached about 100mm^3^, the mice were randomly divided 2 groups of 10 mice each and treated daily by gavage with sunitinib at a dose of 80mg/kg or vehicle (control). The tumor size was assessed every 3 days with caliper measurement, and expressed in mm^3^ using the formula: length×width^2^×0.52. After 15 days of treatment, mice were sacrificed and tumors were snap-frozen in liquid nitrogen. The growth inhibition was measured by the following formula: growth inhibition (%)=1-V/V_0_×100 (V_0_=volume of tumors in control group; V=volume of tumors in sunitinib-treated group).

### Tumor metastasis

Three mice were injected subcutaneously in the right flank with LL/2-P or LL/2-R cells, respectively. After 40 days, mice were sacrificed and lungs were examined, embedded in paraffin, stained for hematoxylin and eosin and photographed using an Olympus BX51 microscope.

### Statistical analysis

Data are presented as means ± SD. Statistical differences were examined by Student’s t test. A P value less than 0.05 is considered to be statistically significant.
